# Genome-wide association study for feed efficiency and growth traits in U.S. beef cattle

**DOI:** 10.1186/s12864-017-3754-y

**Published:** 2017-05-18

**Authors:** Christopher M. Seabury, David L. Oldeschulte, Mahdi Saatchi, Jonathan E. Beever, Jared E. Decker, Yvette A. Halley, Eric K. Bhattarai, Maral Molaei, Harvey C. Freetly, Stephanie L. Hansen, Helen Yampara-Iquise, Kristen A. Johnson, Monty S. Kerley, JaeWoo Kim, Daniel D. Loy, Elisa Marques, Holly L. Neibergs, Robert D. Schnabel, Daniel W. Shike, Matthew L. Spangler, Robert L. Weaber, Dorian J. Garrick, Jeremy F. Taylor

**Affiliations:** 1 Department of Veterinary Pathobiology, Texas A&M University, College Station, 77843 USA; 2 Department of Animal Science, Iowa State University, Ames, 50011 USA; 3 Department of Animal Sciences, University of Illinois, Urbana, 61801 USA; 4 Division of Animal Sciences, University of Missouri, Columbia, 65211 USA; 5 Informatics Institute, University of Missouri, Columbia, 65211 USA; 6 USDA, ARS, US Meat Animal Research Center, Clay Center, 68933 USA; 7 Department of Animal Sciences, Washington State University, Pullman, 99164 USA; 8 GeneSeek a Neogen Company, Lincoln, 68521 USA; 9 Department of Animal Science, University of Nebraska, Lincoln, 68583 USA; 10 Department of Animal Sciences and Industry, Kansas State University, Manhattan, 66506 USA; 11 Institute of Veterinary, Animal and Biomedical Sciences, Massey University, Palmerston North, New Zealand

**Keywords:** GWAS, QTL, Feed efficiency and growth, Beef Cattle

## Abstract

**Background:**

Single nucleotide polymorphism (SNP) arrays for domestic cattle have catalyzed the identification of genetic markers associated with complex traits for inclusion in modern breeding and selection programs. Using actual and imputed Illumina 778K genotypes for 3887 U.S. beef cattle from 3 populations (Angus, Hereford, SimAngus), we performed genome-wide association analyses for feed efficiency and growth traits including average daily gain (ADG), dry matter intake (DMI), mid-test metabolic weight (MMWT), and residual feed intake (RFI), with marker-based heritability estimates produced for all traits and populations.

**Results:**

Moderate and/or large-effect QTL were detected for all traits in all populations, as jointly defined by the estimated proportion of variance explained (PVE) by marker effects (PVE ≥ 1.0%) and a nominal *P*-value threshold (*P* ≤ 5e-05). Lead SNPs with PVE ≥ 2.0% were considered putative evidence of large-effect QTL (*n* = 52), whereas those with PVE ≥ 1.0% but < 2.0% were considered putative evidence for moderate-effect QTL (*n* = 35). Identical or proximal lead SNPs associated with ADG, DMI, MMWT, and RFI collectively supported the potential for either pleiotropic QTL, or independent but proximal causal mutations for multiple traits within and between the analyzed populations. Marker-based heritability estimates for all investigated traits ranged from 0.18 to 0.60 using 778K genotypes, or from 0.17 to 0.57 using 50K genotypes (reduced from Illumina 778K HD to Illumina Bovine SNP50). An investigation to determine if QTL detected by 778K analysis could also be detected using 50K genotypes produced variable results, suggesting that 50K analyses were generally insufficient for QTL detection in these populations, and that relevant breeding or selection programs should be based on higher density analyses (imputed or directly ascertained).

**Conclusions:**

Fourteen moderate to large-effect QTL regions which ranged from being physically proximal (lead SNPs ≤ 3Mb) to fully overlapping for RFI, DMI, ADG, and MMWT were detected within and between populations, and included evidence for pleiotropy, proximal but independent causal mutations, and multi-breed QTL. Bovine positional candidate genes for these traits were functionally conserved across vertebrate species.

**Electronic supplementary material:**

The online version of this article (doi:10.1186/s12864-017-3754-y) contains supplementary material, which is available to authorized users.

## Background

Feed efficiency analyses for beef cattle and other important food animals generally seek to relate measures of feed intake with measures of animal productivity (i.e., agricultural input versus output). Relevant to beef cattle production, and particularly among feedlot operations, the most costly component of the production process is feed, with feed-based expenditures during certain stages of production representing more than 80% of the total costs [1]. The overall economic importance of feed efficiency in beef cattle was initially recognized more than 40 years ago [2, 3], with modern improvements in the efficiency of feed utilization expected to yield positive economic returns across many facets of the beef industry [1, 4]. Moreover, an estimated annual cost savings exceeding $1 billion U.S. dollars could likely be achieved by increasing the efficiency of feed utilization by 10% in U.S. beef cattle alone [5].

Traditional measures of feed efficiency, such as the ratio of feed consumed to observed body weight gain (i.e., feed conversion ratio), are likely to be very successful in selecting for increased growth rates in beef cattle (i.e., increased producer income), but selection for enhanced weaning or yearling weights may also lead to increased costs associated with maintaining larger mature cows (i.e., increased nutrient requirements and feed costs; increased calving difficulty due to larger birth weights, etc) [4, 6, 7]. An alternative measure that has gained in popularity among livestock species is Residual Feed Intake (RFI), with this trait often defined as the difference between an animal’s observed and expected feed intake in relation to the animal’s body weight and growth rate during a specified feeding period (for review see [2, 4, 8–14]). The primary advantages of using RFI to estimate or measure feed efficiency include its phenotypic independence from daily gain as well as the traits used to calculate RFI, with previous heritability estimates among cattle populations ranging from 0.08 to 0.49 [8–10, 12, 13], thereby making RFI a preferred measure for dissecting the underlying biology related to feed efficiency, and for enabling genomic selection [13–15]. Given the economic importance of genomic selection for feed efficiency and growth traits in beef cattle, genome-wide association studies (GWAS) and/or linkage studies have now been performed (for review see [11, 16–20]), with the advent of the Illumina Bovine SNP50 Assay [21], and thereafter, the Illumina Bovine HD Assay [22] directly enabling most of these studies. However, a need remains to thoroughly investigate quantitative trait loci (QTL) associated with RFI as well as other feed intake and growth traits among U.S. beef cattle populations. Therefore, we used a single marker approach with relationship matrix and variance component analysis to map QTL associated with feedlot RFI, average daily dry matter intake (DMI; lb/d), average daily gain on feed (ADG; lb/d), and mid-test metabolic body weight (MMWT; lb^0.75^) in U.S. Angus, Hereford, and SimAngus (Simmental × Angus) beef cattle [13], with corresponding heritability estimates produced for all investigated traits. Thereafter, we investigated whether QTL discovered using the Illumina BovineHD markers (hereafter 778K) were also detectable using the Illumina BovineSNP50 Assay (hereafter 50K), which is important for modern genomic selection programs predicated on historic 50K analyses, and also compared the marker-based heritability estimates produced by the two arrays. Finally, we evaluated our 778K results within the context of the established GWAS literature, and found some positional candidate genes underlying bovine feed efficiency and/or growth related QTL to be conserved across divergent food-animal species, with a tangible proportion of our bovine GWAS results also overlapping with specific loci implicated in studies of obesity or metabolic syndrome, diabetes, and insulin resistance among humans and/or mice. The results of this study are immediately useful for enabling genomic selection in beef cattle, but also suggest that domestic cattle may be relevant models for some human biomedical studies.

## Results and discussion

### Heritability estimates for RFI, DMI, ADG, and MMWT

Using a genomic relationship matrix [23] that was normalized using Gower’s centering approach [24, 25], to yield a sampling variance of 1.0, we estimated the pseudo-heritability [24, 25] [(*i. e*., *h*_*a*_^2^ = σ_*a*_^2^/(σ_*a*_^2^ + σ_*e*_^2^); also represented as: *h* ^2^ = V_A_/(V_A_ + V_E_)] for all investigated feed efficiency and growth traits (RFI, DMI, ADG and MMWT) in U.S. Angus, Hereford, and SimAngus cattle, thereby representing a major segment of the commercial U.S. beef industry (Table 1). The pseudo-heritability, as previously defined [24, 25], is the proportion of phenotypic variance that is explained by the marker-based genomic relationship matrix [23]. We used both the 50K and the 778K marker sets to construct relationship matrices [23]. Pseudo-heritability estimates obtained using the 778K markers ranged from 0.18 to 0.60 across the investigated traits for the three beef cattle populations, with individual ranges of: RFI = 0.20–0.49; DMI = 0.18–0.46; ADG = 0.21–0.37; MMWT = 0.47–0.60 (Table 1). These estimates are similar to those previously reported [13, 16, 17, 26], and are strongly correlated (Angus, all traits, *r* = 0.81; Hereford, all traits, *r* = 0.99; SimAngus, all traits, *r* = 0.77) with previously obtained heritability estimates for the same populations [13]. An *in silico* reduction of the Illumina marker density from 778K to 50K (see Methods) for all populations yielded similar pseudo-heritability estimates across all traits (*r* > 0.99, all traits; see Table 2), suggesting that either SNP platform was suitable for marker-based heritability estimation. Across all populations (i.e., Angus, Hereford, and SimAngus), pseudo-heritability estimates for feed efficiency and growth traits were highest among the Hereford beef cattle (Tables 1 and 2), which is likely due to a small number of contemporary groups and a uniform feeding regiment (see Methods) [13]. In contrast, both the Angus and SimAngus populations were used in nutritional trials, with each population possessing large numbers of contemporary groups, as previously described [13]. Nevertheless, moderate to high pseudo-heritability estimates obtained for feed efficiency and growth traits, particularly RFI and MMWT, further support the expectation of positive economic gains resulting from the implementation of genomic selection for feed efficiency traits in U.S. beef cattle, as evaluated using both multi-marker Bayesian models [13] and the single marker approaches [24, 25] applied in this study.

**Table 1:** Variance component analysis with pseudo-heritability estimates (*h*_*a*_^2^ = σ_*a*_^2^/(σ_*a*_^2^ + σ_*e*_^2^); see references [24, 25]) representing the proportion of phenotypic variance explained by the 778K marker-based relationship matrix [23] for feed efficiency and component traits in U.S. beef cattle

	Angus			Hereford			SimAngus		
Trait	h^2^	V_A_	V_E_	h^2^	V_A_	V_E_	h^2^	V_A_	V_E_
RFI (lb/d)	0.20	0.5821	2.3857	0.49	3.2584	3.4114	0.40	1.4600	2.2300
DMI (lb/d)	0.18	0.9464	4.3881	0.46	4.3725	5.1484	0.26	1.4445	4.0500
ADG (lb/d)	0.21	0.0588	0.2273	0.28	0.1120	0.2862	0.37	0.0860	0.1440
MMWT (lb^0.75^)	0.47	98.990	113.01	0.60	359.12	234.74	0.52	46.643	42.204

**Table 2:** Variance component analysis with pseudo-heritability estimates (*h*_*a*_^2^ = σ_*a*_^2^/(σ_*a*_^2^ + σ_*e*_^2^); see references [24, 25]) representing the proportion of phenotypic variance explained by the 50K marker-based relationship matrix [23] for feed efficiency and component traits in U.S. beef cattle

	Angus			Hereford			SimAngus		
Trait	h^2^	V_A_	V_E_	h^2^	V_A_	V_E_	h^2^	V_A_	V_E_
RFI (lb/d)	0.20	0.5813	2.3865	0.49	3.2749	3.3685	0.37	1.3538	2.3228
DMI (lb/d)	0.17	0.9033	4.4300	0.44	4.1279	5.3371	0.25	1.3792	4.1029
ADG (lb/d)	0.19	0.0539	0.2318	0.27	0.1050	0.2900	0.38	0.0868	0.1425
MMWT (lb^0.75^)	0.44	92.144	119.23	0.57	336.74	253.87	0.52	46.360	42.300

### EMMAX GWAS for RFI in U.S. Beef Cattle

**Fig. 1:**
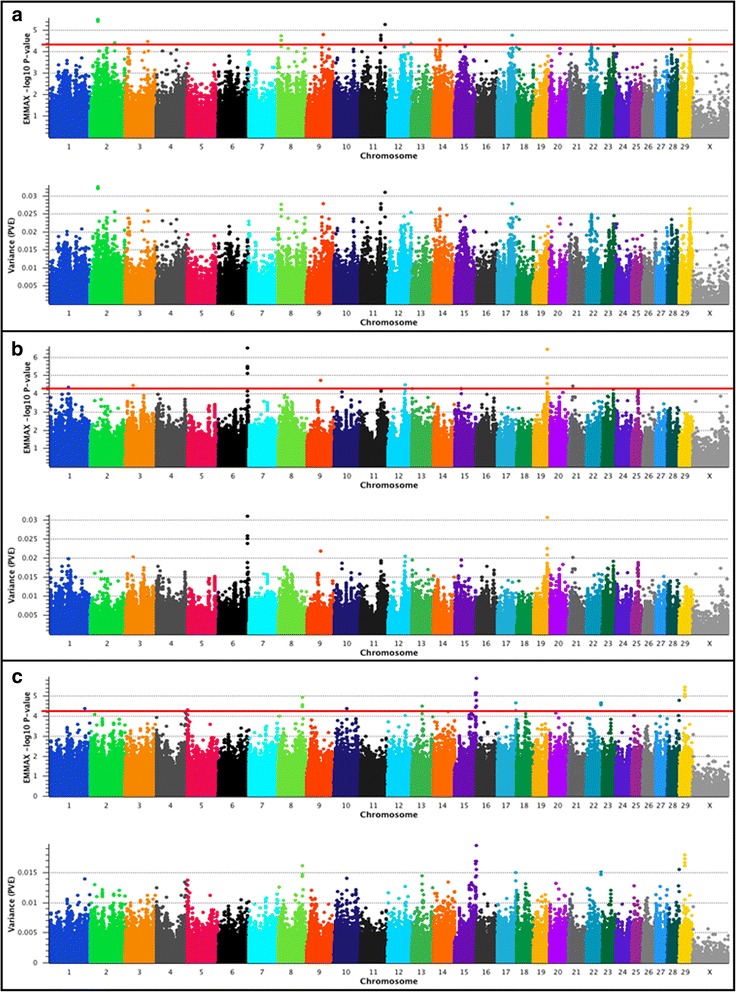
Residual feed intake (RFI) QTL. The top pane of each composite panel reflects a Manhattan plot with EMMAX –log_10_ *P*-values for Illumina 778K markers, whereas the bottom pane depicts the estimated proportion of variance explained (PVE) by marker effects. Lead and supporting SNPs for moderate (1.0% < PVE < 2.0%) or large-effect QTL (PVE ≥ 2.0%) with *P* ≤ 5e-05 and MAF ≥ 0.01 are shown at or above the red line for U.S. Angus (**a**; *n* = 706), Hereford (**b**; *n* = 846), and SimAngus (**c**; *n* = 1217) beef cattle. The pseudo-autosomal region of BTAX is not depicted. A summary of all markers meeting the nominal significance level and MAF cutoff are presented in Additional File 1. Bovine 778K QTL criteria are described in Methods

The results of our Illumina 778K single-marker mixed model analyses for RFI are shown in Fig. 1. Statistical evidence for moderate or large effect QTL was observed for all populations [13, 24, 25]. Lead SNPs (i.e., the most strongly associated SNPs within a QTL region) with estimated proportion of variance explained (PVE; phenotypic) [24, 25] ≥ 2.0% were considered putative evidence of large-effect QTL, whereas lead SNPs with PVE ≥ 1.0% but < 2.0% were considered putative evidence for moderate-effect QTL. Evaluation of all markers meeting the minimum Wellcome Trust significance threshold (*P* < 5e-05) [27] across all three populations revealed markers with PVE that ranged from 1.4% − 3.3% (Additional file 1). A summary of the largest effect QTL detected for RFI is provided in Table 3.

**Table 3:** Summary of the largest effect QTL detected for RFI in U.S. Angus, Hereford, and SimAngus beef cattle

RFI	Position (Chr_Mb)	-log_10_ *P*-value	Regression Beta	PVE	Proximal Positional Candidate Gene(s)	Scientific Precedence [reference]; organism; trait
Angus	2_30	5.51	0.51	0.033	*XIRP2*	[28]; cattle; feed efficiency and growth
	11_80	4.78	−0.45	0.028	*NT5C1B/RDH1*	NA
	17_58	4.77	−1.01	0.028	*HSPB8*	[29]; human; obesity traits
	8_13	4.74	−0.88	0.028	*LOC104969273/LOC104969276*	NA
	14_27	4.56	−0.43	0.027	*TOX/TRNAC-GCA*	[30]; human; adiposity
	29_40	4.55	0.47	0.027	*DDB1/DAK*	[31–35]; human, chicken, duck, pig; cell cycle, feed efficiency and growth
	12_91	4.39	1.48	0.025	*ADPRHL1/CDC-16*	[36, 37]; chicken, pig; feed efficiency and growth
Hereford	6_113	6.51	1.03	0.031	*RAB28*	[40]; human; obesity-related traits
	19_54	6.45	−0.55	0.031	*DNAH17*	[41]; human; adipogenic differentiation
	3_29	4.45	−0.82	0.020	*PTPN22/RBSBN1*	[27]; human; diabetes, arthritis
	1_72	4.34	0.77	0.020	*DLG1*	[42]; human; glucose uptake
SimAngus	15_84	5.90	−0.42	0.020	*STX3/MRPL16/GIF/TCN1*	[43–46]; human, mice, cattle; obesity, glucose levels, growth
	29_20	5.46	0.58	0.018	*LOC101906936*/*LUZP2*	[47]; human; serum magnesium levels
	15_82	5.16	0.39	0.017	*OR9Q2*	[50]; pig; feed efficiency
	8_97	4.96	−0.56	0.016	*LOC101903458*	NA
	22_57	4.67	−0.56	0.015	*TMEM40*	[11, 49]; cattle; feed efficiency
	13_44	4.51	0.58	0.014	*UCN3/CST7*	[53, 54]; mice, cattle; obesity

Seven large-effect QTL (PVE > 2.0%) distributed across seven autosomes were found in Angus using the 778K data (Table 3). Most of the positional candidate genes either underlying or flanking the detected QTL (*XIRP2*, *HSPB8*, *TOX*/*TRNAC-GCA*, *DDB1*, *DAK*, *ADPRHL1*, *CDC-16*; see Table 3) have previously been associated with feed efficiency and growth in other livestock species (i.e., Angus cattle, broilers, domestic fowl, pigs), with components of obesity in humans, and are involved in the resumption of the human cell cycle following the S-phase checkpoint [28–37]. Moreover, one interesting positional candidate gene (lead SNP located within an intron of *DAK*, 29_40 Mb, Table 3) produces the only known enzymatic source of riboflavin 4′, 5′-phosphate (cyclic flavin mononucleotide or FMN) [32], which acts as an electron acceptor in the oxidative metabolism of carbohydrates, amino acids, and fatty acids, but can also donate electrons to the electron transport chain [38, 39]. This is important because riboflavin is known to be essential for energy production (i.e., via oxidative phosphorylation), growth, and development in a variety of species, including humans and livestock [32–35, 38, 39]. Additionally, because riboflavin depletion interferes with the normal progression of the cell cycle [38], the proximity of *DDB1* to *DAK* (29_40 Mb; Table 3) is also quite interesting considering that *DDB1* contributes to the checkpoint recovery process, and the resumption of the human cell cycle [31]. Among the seven large-effect RFI QTL detected in Angus, four (i.e., 2_30 Mb, 11_80 Mb, 14_27 Mb, 29_40 Mb; Table 3) possessed lead SNPs with MAFs ≥ 0.26, while three (i.e., 17_58 Mb, 8_13 Mb, 12_91 Mb; Table 3) possessed minor alleles at lower frequencies (0.01 < MAF < 0.06).

A difference between the present study and a Bayesian analysis (i.e., multi-marker 1 Mb windows) recently reported for the same Angus population [13] was our use of only 706 Angus cattle (See Methods) for the investigation of RFI QTL. We limited our sample size for the RFI GWAS because many of the available Angus cattle were missing information about their age (i.e., birth dates), which negatively impacted our RFI variance component analysis using EMMAX [24, 25] (See Methods). Additionally, the 706 Angus cattle used to investigate RFI QTL in this study were all fed a uniform diet (See Methods). To determine whether proximally similar genomic regions (i.e., within 1 Mb) would be detected using both of the Illumina SNP assays routinely used for QTL discovery (i.e., 50K and 778K), we conducted a second Angus RFI GWAS by rerunning EMMAX [24, 25] after reducing the marker density from 778K to 50K. Subsequently, we observed that only five of the seven largest-effect Angus RFI QTL detected in the 778K analysis (2_30 Mb; 8_13 Mb; 14_27 Mb; 29_40 Mb; 12_91 Mb; Table 3) were proximally replicated (i.e., within ≤ 1 Mb) among the top 100 ranked 50K markers (i.e., with *P* ≤ 5e-05 and/or PVE ≥ 1.0%). Moreover, the 50K lead SNPs (i.e., 8_13 Mb, 14_27 Mb, 29_40 Mb) were physically proximal to the positions of the 778K lead SNPs, supporting the prioritization of the same positional candidate genes as found in the 778K analysis (Table 3).

Among U.S. Hereford cattle, analysis of RFI using the 778K genotypes revealed evidence for four large-effect QTL (PVE Range: 2.0% − 3.1%; Table 3) distributed across four autosomes (6_113 Mb; 19_54 Mb; 3_29 Mb; 1_72 Mb; see Table 3). Evaluation of the positional candidate genes implicated by our 778K analysis (*DNAH17*, *RAB28*, *DLG1*) revealed established associations with human obesity traits [40], adipogenic differentiation [41], type 1 diabetes and rheumatoid arthritis [27], and glucose uptake in mouse cells [42] (see Table 3). Notably, our single-marker approach replicated the Hereford large-effect RFI QTL recently described on BTA19 (i.e., 19_54 Mb; Table 3) using a Bayesian multi-marker 1 Mb window approach [13], but provided positional candidate gene refinement, as the lead SNP in our analysis lies within an intron of *DNAH17*. Relevant to the potential contribution of these loci to selection, the MAFs for the lead SNPs defining the largest-effect RFI QTL detected in Hereford (i.e., 6_113 Mb; 19_54 Mb; 3_29 Mb; 1_72 Mb; Table 3) were moderate to high (MAF Range = 0.07 – 0.35), with the highest MAF corresponding to the QTL detected on BTA19. Following a reduction in marker content to 50K, we conducted a second Hereford RFI GWAS (See Methods) using EMMAX [24, 25] to determine whether the same QTL could be detected using the lower density array. Two of the four large-effect Hereford RFI QTL detected in the 778K analysis (i.e., 6_113 Mb, 19_54 Mb; Table 3) were replicated among the 100 top-ranked markers associated in the 50K GWAS. Moreover, the two largest-effect Hereford RFI QTL replicated via 50K analysis were defined by lead SNPs that were either identical (i.e., 6_113 Mb) or physically proximal to the 778K lead SNPs (i.e., 19_54 Mb; Table 3), thereby supporting the prioritization of the same positional candidate genes.

For U.S. SimAngus cattle, our investigation of RFI using the 778K genotypes produced statistical evidence for one large-effect (PVE ≥ 2.0%) and five moderate-effect (1.0% < PVE < 2.0%) QTL distributed across five autosomes (Table 3). Evaluation of the top six SimAngus RFI QTL signals again revealed positional candidate genes that had previously been implicated in the modulation of traits related to obesity, diabetes, growth, and feed-efficiency (Table 3; Additional file 1). The lead SNP for the largest effect QTL (i.e., *rs135481840*; 15_84 Mb, Table 3) lies within an intergenic region between *STX3*, *GIF* and two adjacent genes (i.e., ncRNA *LOC101907671, TCN1*). Notably, *STX3* is known to underlie a mouse QTL for serum glucose levels [43] and human studies have shown *STX3* to be upregulated in obese subjects following physical exercise [44]. Moreover, both *GIF* and *TCN1* are vitamin B_12_ (i.e., cobalamin) binding proteins and the mean body-weight gains for calves treated with vitamin B_12_ have previously been observed to be superior to those that were untreated during the first 30 weeks of a feeding trial [45]. As was also found for our Hereford RFI QTL scan, a mitochondrial ribosomal protein gene (*MRPL16*) lies within 1 Mb of the largest-effect SimAngus RFI QTL (Table 3), which may be biologically relevant in view of the proposed relationship between mitochondrial function and feed efficiency in livestock [46]. Other notable positional candidates (Table 3; Additional file 1) including *LUZP2* (29_20 Mb), *TMEM72* (28_45 Mb), and *TMEM40* (22_57 Mb) have been associated with serum magnesium levels in humans [47] and feed efficiency and growth traits in cattle [11, 48, 49]. However, despite the biological support for the role of *TMEM72* in RFI [48], only a single marker met the significance threshold for this QTL [27], suggesting caution in further considering a role for this putative QTL in selection or genetic evaluation models.

Interestingly, olfactory transduction pathways modulated by olfactory receptors are known to be associated with RFI in pigs [50], which is conceptually concordant with our detection of the positional candidate gene *OR9Q2* (15_83 Mb; Table 3). We also observed that the largest-effect QTL region detected for RFI in SimAngus (15_84 Mb, Table 3) was flanked by eight olfactory receptor or receptor-like genes (≤0.52 Mb from the lead SNP *rs135481840*). An evaluation of all of the genomic regions that met the minimum significance threshold [27] (PVE Range = 1.4% – 2.0%) in the analysis of the 778K genotypes revealed that multiple markers supported the six largest-effect SimAngus RFI QTL (Table 3), but that several other biologically relevant positional candidate genes were supported by only a single significant marker (Additional file 1). Positional candidate genes corresponding to several of these putative moderate-effect QTL (PVE < 2.0%) have previously been associated with lipid metabolism and intramuscular fat deposition in chickens (*YWHAH*) [51], obesity traits in mice and humans (*CST7*, *ADAM10*) [52, 53] and RFI in cattle (*CST7*) [54] (Additional file 1). Analysis of the 50K genotype set for RFI in SimAngus resulted in only two markers meeting the minimum significance threshold [27], and identified only one (22_57 Mb) of the six QTL that were detected in the 778K analysis (Table 3). However, among the 100 top-ranked 50K markers, we found evidence supporting three of the largest-effect SimAngus RFI QTL that were detected in the 778K analysis (i.e., ≤ 1 Mb from 22_57 Mb, 8_97 Mb, 15_83 Mb; Table 3).

### EMMAX GWAS for DMI in U.S. Beef Cattle

**Fig. 2:**
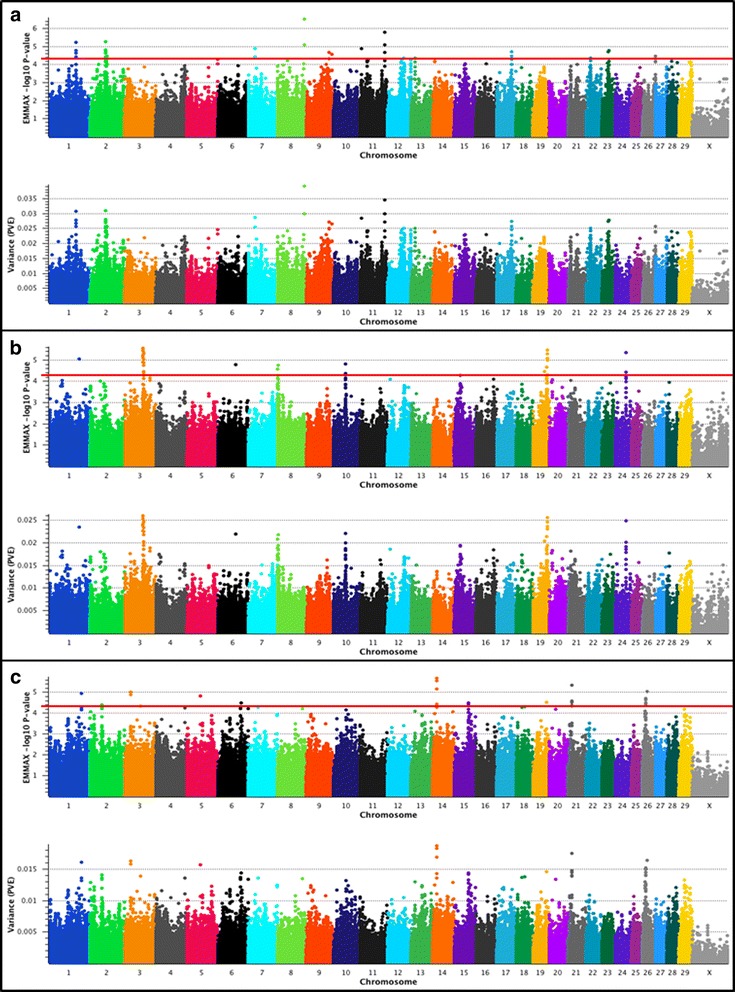
Dry matter intake (DMI) QTL. The top pane of each composite panel reflects a Manhattan plot with EMMAX –log_10_ *P*-values for Illumina 778K markers, whereas the bottom pane depicts the estimated proportion of variance explained (PVE) by marker effects. Lead and supporting SNPs for moderate (1.0% < PVE < 2.0%) or large-effect QTL (PVE ≥ 2.0%) with *P* ≤ 5e-05 and MAF ≥ 0.01 are shown at or above the red line for U.S. Angus (**a**; *n* = 706), Hereford (**b**; *n* = 846), and SimAngus (**c**; *n* = 1218) beef cattle. The pseudo-autosomal region of BTAX is not depicted. A summary of all markers meeting the nominal significance level and MAF cutoff are presented in Additional File 1. Bovine 778K QTL criteria are described in Methods

Results of the single-marker mixed model analyses using 778K genotypes for DMI are shown in Fig. 2. Statistical evidence for moderate or large-effect DMI QTL was again observed for all of the investigated populations [13, 24, 25]. An evaluation of all markers meeting the minimum significance threshold (*P* < 5e-05) [27] across all three populations revealed estimates of PVE that ranged from 1.4% − 3.9% (Additional file 1). Summary data for the largest effect QTL detected for DMI are provided in Table 4.

**Table 4:** Summary of the largest effect QTL detected for DMI in U.S. Angus, Hereford, and SimAngus beef cattle

DMI	Position (Chr_Mb)	-log_10_ *P*-value	Regression Beta	PVE	Proximal/Putative Candidate Gene(s)	Scientific Precedence [reference]; organism; trait
Angus	8_107	6.53	−2.91	0.039	*LOC101902746/TNC*	[55]; cattle; feed efficiency
	11_97	5.81	2.47	0.034	*MVB12B/LMX1B*	[56]; human; obesity traits
	2_63	5.26	−0.74	0.031	*TMEM163/MGAT5*	[58, 59, 62]; human, mice; diabetes, nutrient sensing
	1_106	5.25	0.67	0.031	*LOC782622/LOC785220*	NA
	7_27	4.89	−2.09	0.029	*SLC12A2*	[11]; cattle; feed efficiency
	11_7	4.88	0.89	0.028	*IL1R2*	[55]; cattle; feed efficiency
	17_57	4.72	0.78	0.027	*CUX2*	[57]; human; diabetes
	9_90	4.68	−0.57	0.027	*ESR1*	[60, 61]; pig, human; feed efficiency, diabetes, obesity
Hereford	3_70	5.56	0.76	0.026	*TYW3/CRYZ*	[67]; human; insulin resistance
	19_55	5.48	0.79	0.026	*MGAT5B*	[68]; mice; glycosylation
	24_41	5.35	−0.98	0.025	*RAB12*	[69]; mice; cellular nutrient sensing
	3_73	5.35	0.98	0.025	*NEGR1*	[70–72]; humans, rats; feed intake, obesity
	19_57	5.09	−0.92	0.024	*RAB37*	[73]; mice; insulin release
	10_47	4.81	0.91	0.022	*CA12/LACTB*	[74, 75]; humans, mice; pH balance, adiposity
SimAngus	14_17	5.68	−1.05	0.019	*NDUFB9/RNF139/MTSS1/SQLE*	[28, 82–85]; cattle, human, mice; feed efficiency, diabetes, obesity
	21_16	5.36	−0.49	0.018	*SV2B*	[86, 87] human; insulin secretion and weight loss
	3_22	5.01	−0.49	0.016	*LOC104971520/CHD1L/LOC788724/FMO5*	[88]; mice; obesity and diabetes
	26_14	4.70	0.50	0.015	*IDE/CYP26A1*	[89–91]; humans, mice; insulin degradation, diabetes, obesity
	15_58	4.50	0.59	0.014	*CAPN5/MYO7A*	[92, 93]; cattle, chicken; meat tenderness; abdominal fat
	6_91	4.49	−0.45	0.014	*CXCL1*	[94]; mice; diet-induced obesity
	2_47	4.39	−0.50	0.014	*LYPD6*	[55]; cattle; feed efficiency

Among Angus, an investigation of the largest-effect DMI QTL (Table 4) detected using the 778K genotypes revealed at least seven biologically relevant positional candidate genes (Table 4) that have previously been associated with feed efficiency, diabetes, and obesity traits in livestock and humans, respectively [11, 55–62]. For example, *TNC* (8_107 Mb; Table 4) has previously been reported to be differentially expressed between low- and high-RFI Angus cattle [55], and *LMX1B* (11_97 Mb; Table 4) has recently been implicated in human age-related obesity, with obese subjects displaying decreased methylation with age [56]. Across the eight largest-effect DMI QTL revealed by the 778K markers that met the minimum significance threshold [27] (PVE ≥ 2.0%; Table 4), those located on BTA2 (2_63 Mb), BTA1 (1_106 Mb), BTA11 (11_7 Mb), and BTA9 (9_90 Mb) were individually supported by the greatest numbers of markers (Additional file 1). Moreover, among the largest-effect Angus DMI QTL (Table 4), three positional candidate genes (*CUX2*, *TMEM163*, *ESR1*) have been associated with diabetes in humans [57–59, 61], while four (*TNC*, *SLC12A2*, *IL1R2*, *ESR1*) have been associated with aspects of feed efficiency and growth in cattle [11, 55] or adiposity in pigs [60]. Further investigation of all Angus DMI QTL regions detected in the 778K analysis revealed several additional candidate genes of interest, including *MGAT5* (2_63 Mb). Notably, Mgat5^-/-^ null mice were previously shown to experience diminished glycemic response to exogenous glucagon, and increased insulin sensitivity [62]. Moreover, positional candidate genes associated with feeding behavior and growth in pigs (*GATA3*, *GLRX3*) [15, 63, 64] as well as obesity traits in mice (*IL17A*) [65, 66] were also located within the Angus DMI QTL regions. Similar to our RFI analyses, differences between the results presented here and those previously reported for the same Angus population [13] primarily stem from our use of 706 Angus cattle (*n* = 706 with age data and a uniform diet; See Methods) for the investigation of DMI QTL. The MAFs for all lead SNPs defining the eight largest-effect DMI QTL in Angus ranged from 0.01 (BTA8_107 Mb) to 0.49 (BTA9_90 Mb), with five of the eight QTL having lead SNPs with MAFs ≥ 0.11. We next analyzed DMI using 50K genotypes and found evidence for three of the eight largest-effect QTL detected by 778K analysis (11_97 Mb; 17_57 Mb; 9_90 Mb; Table 4). Moreover, among the 100 top-ranked 50K markers, we found evidence for the replication of two additional Angus DMI QTL detected in the 778K analysis (2_63 Mb; 7_27 Mb; Table 4).

Evaluation of the largest-effect DMI QTL detected for Hereford in the 778K analysis revealed at least five biologically relevant positional candidate genes (*TYW3*/*CRYZ*, *MGAT5B, RAB12*, *RAB37*) that may harbor genetic variation influencing aspects of feed intake, including appetite (Table 4). The genomic region harboring *TYW3* and *CRYZ* (3_70 Mb; Table 4) has previously been associated with insulin resistance (resistin) in humans [67], while Mgat5^-/-^ null mice exhibit increased insulin sensitivity [62]. The evolutionarily related gene family member and positional candidate *MGAT5B* (*MGAT5* isozyme B or *GnT-VB*; 19_55 Mb) underlies the second largest-effect QTL detected for DMI in Hereford (Table 4). *MGAT5* and *MGAT5B* are both known to be involved in the biosynthesis of N-glycans [62, 68], and both genes have been implicated as positional candidates for large-effect DMI QTL in Angus and Hereford (Table 4). Moreover, the positional candidate genes *RAB12* (24_41 Mb), *NEGR1* (3_73 Mb), *RAB37* (19_57 Mb), and *CA12*/*LACTB* (10_47 Mb) have also been associated with autophagy related to cellular nutrient sensing in mice [69], feed intake and/or obesity traits in humans and rats [70–72], insulin release in mice [73], hyponatremia in humans with loss of appetite and poor weight gain [74], and adiposity in humans [75] (Table 4). Importantly, the biochemical roles and pathway relationships between insulin and glucagon with respect to food intake, satiety, and body weight have been reported and reviewed, with both insulin and glucagon normally acting to reduce meal size [76, 77]. The large-effect Hereford DMI QTL detected on BTA3 (3_70 Mb) is generally compatible with a recent Bayesian analysis of these data which utilized 1 Mb windows [13], but herein, we used single markers to identify specific positional candidate genes that were most proximal to the putative QTL peak(s), as defined by the lead SNP (Table 4). Investigation of all Illumina 778K markers meeting the minimum significance threshold [27] (PVE Range = 2.0% – 2.6%) provided additional support for *TYW3*/*CRYZ*, *MGAT5B*, *RAB12*, *NEGR1,* and *RAB37*, but also revealed several other biologically relevant positional candidate genes with less marker-based support (i.e., only one SNP meeting the significance threshold; Additional file 1). For example, *CORIN* (BTA6_68 Mb) has previously been associated with body weight and obesity traits in mice [78, 79], while *DNAH17* (BTA19_54 Mb), *ANXA10* (BTA8_1 Mb) and *AADAT* (8_2 Mb), have all been associated with aspects of feed efficiency and growth in either beef cattle or chickens [13, 80, 81] (Additional file 1). The MAFs for all lead SNPs defining the six large-effect DMI QTL in Hereford ranged from 0.15 (BTA24_41 Mb; BTA3_73 Mb; 19_57 Mb; 10_47 Mb) to 0.32 (BTA3_70 Mb). In the analysis of the 50K genotype set three markers met the minimum significance threshold [27], and two (19_57 Mb, *RAB37*; 3_73 Mb, *NEGR1*) supported large-effect QTL detected in the 778K analysis (i.e., within 1 Mb; Table 4). Moreover, SNPs proximal to all of the largest-effect Hereford DMI QTL detected in the 778K analysis (Table 4) were observed among the 100 top-ranked 50K markers.

Analysis of DMI for the SimAngus cattle using the 778K genotypes revealed seven moderate-effect QTL (1.0% < PVE < 2.0%) with at least ten biologically relevant positional candidate genes (*NDUFB9*, *RNF139*, *MTSS1*, *SV2B*, *FMO5*, *IDE*, *CYP26A1*, *CAPN5*, *MYO7A*, *CXCL1*, *LYPD6*, see Table 4). The largest-effect DMI QTL region (14_17 Mb; Table 4) included genes that have previously been associated with several aspects of feed efficiency (*NDUFB9*, *RNF139*) in beef cattle [28, 82] and Type 2 diabetes (*MTSS1*) in humans [83]. Moreover, all of the positional candidate genes near the largest-effect DMI QTL (14_17 Mb; Table 4) were also immediately flanked by *SQLE* (14_16.7 Mb), a cholesterol biosynthesis enzyme previously implicated as a primary positional candidate for a large-effect obesity QTL in mice [84, 85]. Proximal to the second largest-effect SimAngus DMI QTL, we detected *SV2B* (21_16 Mb; Table 4), which may be involved in aspects of regulated insulin secretion [86], and has been associated with human weight-loss across a diverse array of dietary regimes [87]. It should also be noted that previous studies also support the involvement of *FMO5*, *IDE*, *CYP26A1*, *CAPN5*, *MYO7A*, and *CXCL1* (Table 4) in the manifestation of obesity traits in multiple vertebrate species (human, mice, poultry), in the onset of diabetes in humans, and/or in aspects of beef palatability [88–94]. Interestingly, while the DMI positional candidate gene *MYO7A* (15_57 Mb) has previously been associated with abdominal fat deposition in broilers, another proximal positional candidate gene (*CAPN5*, 15_57 Mb) has been associated with beef tenderness in Nelore cattle [92, 93]. Additionally, the lead SNP defining a DMI QTL on BTA2 (2_47 Mb; Table 4) was proximal to the transcriptional start site of *LYPD6*, which has previously been found to be differentially expressed between low- and high-RFI cattle [55]. An investigation of all 778K markers that met the minimum significance threshold [27] (PVE Range = 1.4% – 1.9%) revealed additional support for the seven moderate-effect DMI QTL (Additional file 1). Mixed model analysis of DMI in SimAngus using the 50K marker set revealed four markers that met the minimum significance threshold [27] and these corresponded to three of the six moderate-effect QTL detected in the 778K analysis (were ≤ 1 Mb from 14_17 Mb, 21_16 Mb, 15_57 Mb; Table 4). Further investigation of the 100 top-ranked 50K markers revealed evidence for the replication of two additional QTL detected in the 778K analysis (i.e., were ≤ 1 Mb from 26_14 Mb and 3_22 Mb; Table 4).

### EMMAX GWAS for ADG in U.S. Beef Cattle

**Fig. 3:**
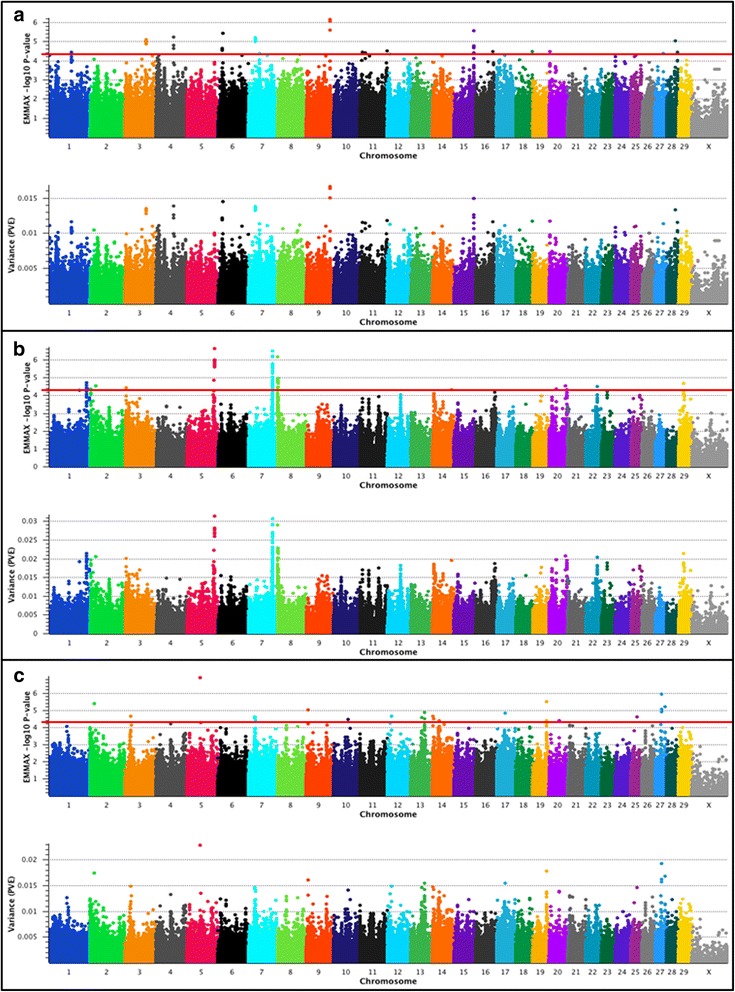
Average daily gain (ADG) QTL. The top pane of each composite panel reflects a Manhattan plot with EMMAX –log_10_ *P*-values for Illumina 778K markers, whereas the bottom pane depicts the estimated proportion of variance explained (PVE) by marker effects. Lead and supporting SNPs for moderate (1.0% < PVE < 2.0%) or large-effect QTL (PVE ≥ 2.0%) with *P* ≤ 5e-05 and MAF ≥ 0.01 are shown at or above the red line for U.S. Angus (**a**; n = 1572), Hereford (**b**; *n* = 849), and SimAngus (**c**; *n* = 1237) beef cattle. The pseudo-autosomal region of BTAX is not depicted. A summary of all markers meeting the nominal significance level and MAF cutoff are presented in Additional File 1. Bovine 778K QTL criteria are described in Methods

Results of the 778K single-marker mixed model analyses for ADG are presented in Fig. 3 and Table 5. Statistical evidence for moderate or large-effect QTL was observed for all of the investigated populations [13, 24, 25] with PVE that ranged from 1.1% − 3.2% (Additional file 1).

**Table 5:** Summary of the largest effect QTL detected for ADG in U.S. Angus, Hereford, and SimAngus beef cattle

ADG	Position (Chr_Mb)	-log_10_ *P*-value	Regression Beta	PVE	Proximal/Putative Candidate Gene(s)	Scientific Precedence [reference]; organism; trait
Angus	9_93	6.16	−0.11	0.017	*NOX3*	[95]; mice, human; insulin resistance
	15_79	5.57	−0.10	0.015	*OR4X1*	[50, 96]; pig, cattle; feed efficiency
	4_69	5.22	0.11	0.014	*SKAP2/LOC101903616*	[97]; human; diabetes
	7_26	5.20	−0.09	0.014	*SLC12A2/ADAMTS19/SLC27A6*	[11, 98, 99]; cattle, human, cattle; feed efficiency, interaction with *IGF2R*, fatty acid composition in bovine milk
	3_82	5.10	−0.10	0.013	*PGMI*	[100]; pig; feed efficiency
	6_16	4.64	0.10	0.012	*PITX2/EGF*	[101, 102]; human, rabbit; adipogenesis, regulated absorption of nutrients
Hereford	5_106	6.64	−0.18	0.031	*FGF6/CCDN2/FGF23*	[103–105]; cattle, human; muscle mass in carcass, cell cycle regulation, insulin resistance and obesity
	7_93	6.51	−0.15	0.031	*ARRDC3*	[106]; human, mice; body mass and energy expenditure
	8_1	6.17	0.17	0.029	*PALLD*	[107]; human; adipocyte size
	8_4	4.96	−0.15	0.023	*GALNTL6*	[108–110]; cattle; feed efficiency and growth
	8_2	4.82	0.13	0.022	*LOC511409/LOC101904351/LOC520638/AADAT*	[81, 111]; chicken, seabass; feed efficiency and growth
	1_144	4.71	−0.45	0.021	*ABCG1/TFF3/TFF2/TFF1/UBASH3A*	[112–114]; human, mice, pig; obesity, glucose tolerance, marbling score
	2_23	4.53	−0.25	0.021	*GPR155/SCRN3/SP9/OLA1*	[50; 116] pig, cattle; feed efficiency; infectious bovine keratoconjuctivitis
SimAngus	27_26	5.93	−0.15	0.019	*GTF2E2*	[117]; human; heart failure
	19_54	5.52	0.10	0.018	*DNAH17/RBFOX3*	[41]; human; adipogenic differentiation
	13_53	4.87	0.16	0.016	*SNORA70/STK35/PDYN/SIRPA*	[118–121]; human, cattle, mouse; diabetes, bovine liver prohormone, mouse feeding behavior and obesity traits, human adiposity
	3_22	4.67	−0.10	0.015	*LOC104971520/CHD1L/LOC788724/FMO5*	[88, 122, 123]; mice, human; obesity and diabetes, cellular proliferation
	7_26	4.61	0.12	0.015	*ADAMTS19/SLC27A6*	[98, 99]; human, cattle; interaction with *IGF2R*, fatty acid composition in bovine milk
	14_7	4.51	−0.26	0.014	*LOC100848525/KHDRBS3*	[124, 125]; cattle; intramuscular fat
	20_39	4.41	0.09	0.014	*RAI14*	[126, 127]; mice; obesity

An investigation of the largest-effect ADG QTL detected in Angus in the 778K analysis revealed at least 9 biologically relevant positional candidate genes (*NOX3*, *OR4X1*, *SKAP2*, *ADAMTS19*, *SLC27A6*, *SLC12A2*, *PGMI*, *PITX2*, *EGF*, Table 5) previously associated with livestock feed efficiency, insulin resistance, Type 1 diabetes, and adipogenesis in humans [11, 50, 95–102]. The largest-effect ADG QTL was detected on BTA9 (9_93 Mb) upstream of *NOX3*, which has previously been shown to modulate palmitate-induced insulin resistance in human hepatic cells [95]. We also detected an ADG QTL on BTA15 (15_79 Mb; *OR4X1*; Table 5), which was located within an olfactory receptor and receptor-like gene cluster. Olfactory receptors have previously been proposed as underlying feed efficiency QTL in pigs [50], and this QTL is proximal to the RFI QTL detected in SimAngus (*OR9Q2*; Table 3) [13]. A moderate- effect ADG QTL detected on BTA4 (4_69 Mb) suggested only a single positional candidate gene (*SKAP2*) that has previously been confirmed to be associated with Type 1 diabetes in humans [97], and additional positional candidate genes underlying Angus ADG QTL on BTA7 (7_26 Mb; *SLC12A2*) and BTA3 (3_82 Mb; *PGMI*) have also been associated with feed efficiency in cattle and pigs, respectively [11, 100]. We identified two positional candidate genes (*PITX2*, *EGF*; Table 5) for the ADG QTL detected on BTA6 (6_16 Mb). While *PITX2* is known to be associated with stem cell commitment to adipogenesis in humans [101], *EGF* has been shown to regulate the absorption of nutrients and electrolytes from the small intestine of rabbits [102]. Evaluation of all genes proximal to the largest-effect ADG QTL detected in Angus revealed at least two additional positional candidate genes on BTA7 (7_26 Mb; *ADAMTS19*, *SLC27A6*; Table 5). Significant synergistic interactions have been reported between human genetic variation in *ADAMTS19* and *IGFR2* [98], whereas genetic variation in *SLC27A6* has been associated with fatty acid composition of bovine milk [99]. The MAFs for all lead SNPs defining the six moderate-effect ADG QTL in Angus ranged from 0.24 (BTA6_16 Mb) to 0.45 (BTA7_26 Mb), with five of the six QTL having lead SNPs with MAFs ≥ 0.25 (Additional file 1). The 50K analysis produced seven markers that met the minimum significance threshold [27], which corresponded to three (15_79 Mb; 3_82 Mb; 6_16 Mb) of the six largest effect QTL detected by the 778K analysis (Table 5). Evidence for the ADG QTL on BTA9 (9_93 Mb) was found among the nine top-ranked markers from the 50K analysis. Notably, the Angus ADG QTL detected on BTA4 (4_69 Mb) and BTA7 (7_26 Mb) in the 778K analysis were not supported by the locations of the top 100 ranked 50K markers.

Analysis of ADG in Hereford cattle using the 778K genotypes revealed at least eight biologically relevant positional candidate genes corresponding to seven large-effect QTL (PVE Range = 2.1% – 3.1%; Table 5). Positional candidate genes proximal to the largest-effect QTL on BTA5 (5_106 Mb; *FGF6*, *FGF23*, *CCND2*; Table 5) have previously been associated with carcass muscle mass in Charolais cattle, blood-based markers for insulin resistance and obesity in humans, and insulin resistance versus sensitivity in human adipose tissue, respectively [103–105]. A large-effect QTL also was detected on BTA7 (7_93 Mb), for which we found one underlying positional candidate gene (*ARRDC3*) that has previously been associated with obesity in humans and mice [106]. Four additional large-effect ADG QTL signals were also detected on BTA8 (8_1 Mb; 8_4 Mb; 8_2 Mb) and BTA1 (1_144 Mb; Table 5). Further evaluations of these QTL revealed positional candidate genes which have previously been associated with human adipocyte size (*PALLD*) [107] as well as feed efficiency and growth traits in cattle (*GALNTL6*) [108–110]; with a recent investigation providing some evidence for bovine copy number variants proximal to *GALNTL6* [110]. Overlapping ADG and DMI QTL were detected on BTA8 (8_1 Mb; 8_2 Mb) in Hereford, with *PALLD* and *AADAT* being positional candidate genes for both traits. Notably, while *AADAT* has previously been associated with feed efficiency and growth traits in both poultry and seabass [81, 111] (Table 5), the BTA8 QTL signals (8_1 Mb; 8_2 Mb) for DMI in Hereford were limited to a single lead SNP underlying each QTL that met the minimum significance threshold [27] (Additional file 1). An investigation of the QTL on BTA1 revealed at least three biologically relevant positional candidate genes (*ABCG1*, *TFF3*, *UBASH3A*; Table 5). The ATP-binding cassette transporter protein encoded by *ABCG1* has unambiguously been shown to possess a major role in adiposity and fat mass growth in humans as well as mice [112], while increased levels of the intestinal protein encoded by *TFF3* improved glucose tolerance in a diet-induced mouse obesity model [113]. Along the same lines, the positional candidate *UBASH3A* has previously been associated with marbling score in pigs [114], and is differentially methylated among peripheral blood leukocytes derived from lean and obese human adolescents [115]. Finally, we also detected a large-effect QTL on BTA2 (2_23 Mb; lead SNP 22.63 Mb; PVE = 2.1%), with at least two relevant positional candidate genes suggested by the lead and supporting SNPs including *GPR155*, which has been associated with RFI in pigs [50], and *OLA1*, which has been associated with the incidence of infectious bovine keratoconjunctivitis in Angus cattle [116]. The results of our 778K analysis are generally concordant with a recent Bayesian analysis that utilized 1 Mb windows to investigate ADG QTL in U.S. Hereford cattle [13]; with our analysis further refining the positional candidate genes for large-effect QTL detected by both analyses on BTA5, BTA7, and BTA8. Moreover, we also provide evidence for additional ADG QTL on BTA8 (8_2 Mb; 8_4 Mb), BTA1 (1_144 Mb), and BTA2 (2_23 Mb) that were not previously detected [13]. MAFs for all lead SNPs defining the six ADG QTL in Hereford ranged from 0.02 (BTA1_144 Mb) to 0.47 (BTA7_93 Mb). Reduction of the 778K marker set to 50K revealed eight markers that met the minimum significance threshold and provided evidence for the replication of QTL on BTA5 (5_106Mb), BTA7 (7_93 Mb), and BTA8 (8_1 Mb). Moreover, while ADG QTL signals detected by 778K analysis on BTA8 (8_4 Mb) and BTA2 (2_23 Mb) were supported by the locations of the top 100 ranked 50K markers, two QTL were not (i.e., 1_144 Mb; 8_2 Mb), indicating an inability to detect all relevant Hereford ADG QTL using the 50K marker set.

Seven moderate-effect ADG QTL (1.0% > PVE < 2.0%) were detected in the 778K SimAngus analysis, with three of these (3_22 Mb; 7_26 Mb; 19_54 Mb; Table 5) predicted to be proximal to, or overlap with, QTL detected for other traits or populations. For example, the SimAngus ADG QTL detected on BTA3 (3_22 Mb; Table 5) was also detected in the SimAngus DMI analysis (Table 4), suggesting either pleiotropy or that independent but proximal causal mutations influence ADG and DMI. Identical lead SNPs were found for both traits in SimAngus, which is concordant with a pleiotropic QTL. Further, the SimAngus ADG QTL detected on BTA7 (7_26 Mb; Table 5) was also detected in the analysis of ADG in Angus (Table 5), and was proximal (≤ 1 Mb) to an Angus DMI QTL (7_27 Mb; Table 4). These results were relatively unsurprising considering the Angus influence within this SimAngus population. While the lead SNPs defining the ADG and DMI QTL (7_26 Mb; 7_27 Mb) were not concordant in Angus, the direction of the SNP effects were concordant (Table 4; Table 5), suggesting either proximal but independent causal mutations, or the inability to accurately estimate a pleiotropic QTL position based on the Angus sample size for DMI (*n* = 706). Finally, the SimAngus ADG QTL detected on BTA19 (19_54 Mb) overlaps with one of the largest-effect QTL detected for RFI in Hereford (Table 3), and was also proximal (≤ 1.71 Mb; 19_55 Mb) to a Hereford DMI QTL (Table 4). Therefore, our analyses of these data suggests the existence of pleiotropic feed efficiency and growth QTL in U.S. beef populations that have not previously been reported [13]. Moreover, the overlap also suggests that positional candidate genes on BTA3 (*CHD1L*, *FMO5*; 3_22 Mb), BTA7 (*ADAMTS19*, *SLC27A6*; 7_26 Mb), and BTA19 (*DNAH17*, *RBFOX3*; 19_54 Mb) may potentially hold biological value beyond selection for ADG in SimAngus cattle (Table 5), as overlapping or proximal genomic regions and corresponding positional candidate genes were identified during our analyses of RFI, DMI, and ADG for all three populations (see Tables 3–5).

Beyond the pleiotropic and multi-breed QTL described above, moderate-effect QTL for ADG were also detected on BTA27 (27_26 Mb), BTA13 (13_53 Mb), BTA14 (14_7 Mb), and BTA20 (20_39 Mb) in SimAngus (Table 5). The QTL explaining the largest proportion of variance in ADG (BTA27_26 Mb) colocalized with the positional candidate gene *GTF2E2*, which has previously been associated with a metabolite (X-11787; hydroxy-leucine or hydroxy-isoleucine) that is predictive of heart failure in African Americans [117]. Additionally, at least three positional candidate genes were identified (*STK35*, *PDYN*, *SIRPA*) for the ADG QTL on BTA13 (13_53 Mb). In particular, *STK35*, which is a nuclear Serine/Threonine kinase, is differentially expressed in the peripheral blood monocytes of Type 1 versus Type 2 diabetes patients [118], while *PDYN* (prodynorphin) is a prohormone that is differentially expressed in the livers of cattle exposed to differing nutritional statuses [119]. *PDYN* knockout affects feeding behavior, fasting weight loss, fat mass, and bone mineral content in mice [120], whereas *SIRPA* expression in human platelets was found to interact with obesity traits such as adiposity in a study of inflammatory proteins and artherosclerosis [121]. In regards to the overlapping SimAngus ADG and DMI QTL (3_22 Mb), *FMO5* (BTA3_22 Mb) has been associated with obesity and diabetes in mice [88] and *CHD1L* is known to influence cellular proliferation [122, 123]. Evaluation of the SimAngus ADG QTL on BTA14 (14_7 Mb) revealed an annotated positional candidate gene (*KHDRBS3*) that is associated with intramuscular fat deposition in cattle [124, 125]. A positional candidate gene (*RAI14*) for the ADG QTL on BTA20 (20_39 Mb), is associated with obesity in mice [126, 127]. Similarly, while the positional candidate gene *DNAH17* (19_54 Mb) has also been associated with aspects of human adipogenesis (adipogenic differentiation) [40], a flanking positional candidate gene for this QTL (*RBFOX3*; Table 5) has been associated with serum urate levels in relation to human BMI [128]. The MAFs for all lead SNPs defining the seven moderate-effect ADG QTL in SimAngus ranged from 0.03 (BTA14_7 Mb) to nearly 0.50 (BTA3_22 Mb, MAF = 0.496), with five of the seven QTL having lead SNPs with MAFs ≥ 0.12 (Additional file 1). After reducing the marker density from 778K to 50K and reanalyzing ADG, five markers met the minimum significance threshold [27], including evidence for QTL replication on BTA20 (20_39 Mb). Among the top 100 ranked 50K markers, we only found evidence for the replication of two additional SimAngus ADG QTL detected in the 778K analysis (27_26 Mb; 7_26 Mb), again underscoring an inability to detect ADG QTL using the reduced marker panel.

### EMMAX GWAS for MMWT in U.S. Beef Cattle

**Fig. 4:**
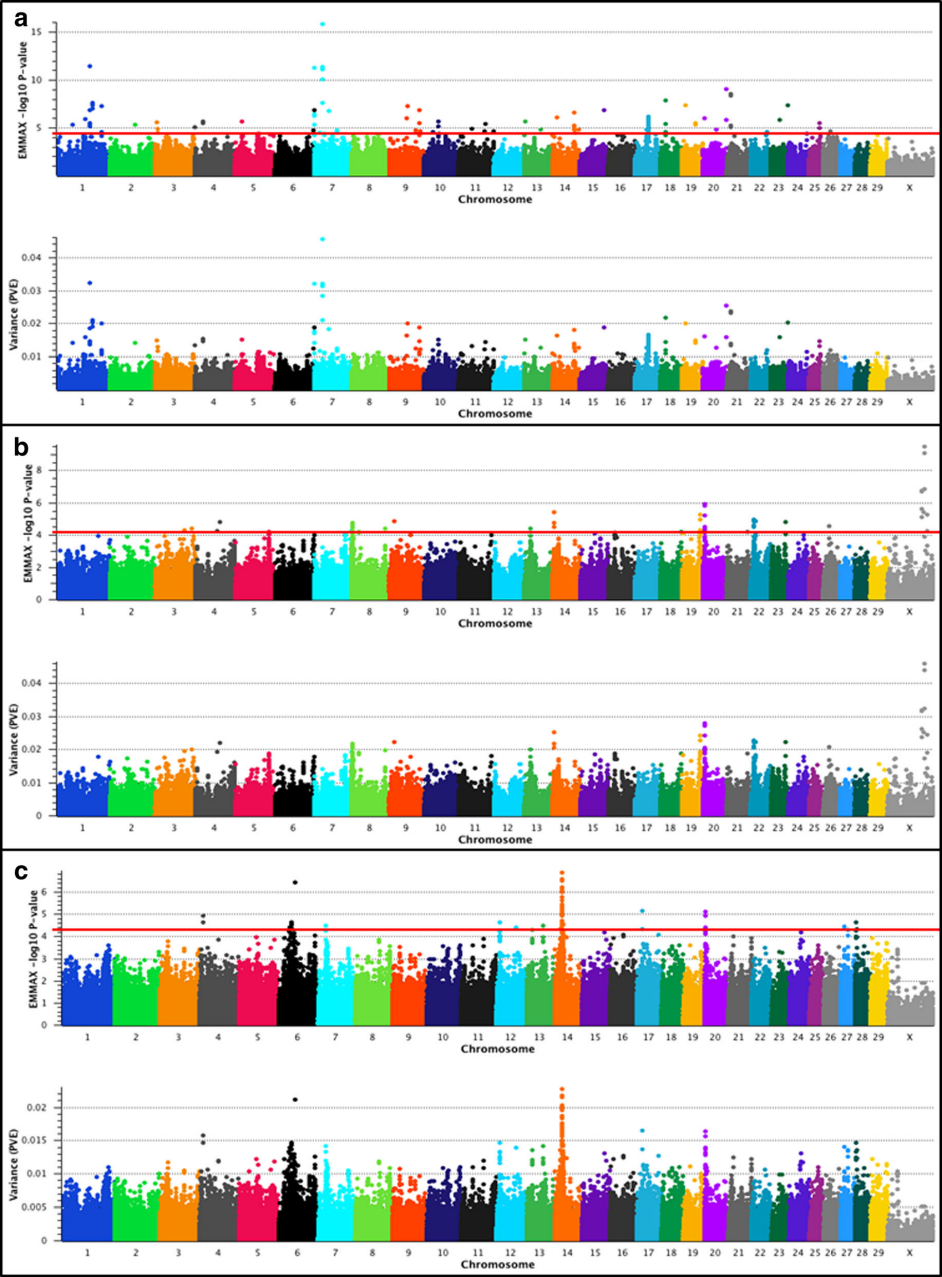
Mid-test metabolic weight (MMWT) QTL. The top pane of each composite panel reflects a Manhattan plot with EMMAX –log_10_ *P*-values for Illumina 778K markers, whereas the bottom pane depicts the estimated proportion of variance explained (PVE) by marker effects. Lead and supporting SNPs for moderate (1.0% < PVE < 2.0%) or large-effect QTL (PVE ≥ 2.0%) with *P* ≤ 5e-05 and MAF ≥ 0.01 are shown at or above the red line for U.S. Angus (**a**; *n* = 1572), Hereford (**b**; *n* = 849), and SimAngus (**c**; *n* = 1238) beef cattle. The pseudo-autosomal region of BTAX is not depicted. A summary of all markers meeting the nominal significance level and MAF cutoff are presented in Additional File 1. Bovine 778K QTL criteria are described in Methods

Results of the 778K single-marker mixed model analyses for MMWT are presented in Fig. 4 and Table 6. Evidence for moderate or large-effect QTL was observed for all of the populations with PVE that ranged from 1.1% − 4.6% (Additional file 1).

**Table 6:** Summary of the largest effect QTL detected for MMWT in U.S. Angus, Hereford, and SimAngus beef cattle

MMWT	Position (Chr_Mb)	-log_10_ *P*-value	Regression Beta	PVE	Proximal/Putative Candidate Gene(s)	Scientific Precedence [reference]; organism; trait
Angus	7_24	15.88	6.86	0.046	*ACSL6*	[13, 129]; cattle, rodents; feed efficiency and growth; diabetic myocardial metabolism
	1_98	11.45	6.08	0.032	*MYNN/ACTRT3/LOC104970973/MECOM*	[130, 131]; human, mice; obesity and diabetes traits
	7_0	11.31	6.97	0.032	*LOC100300983/LOC100125913/LOC101902704/LOC101902742/FLT4/CNOT6*	[134, 135]; human; cell growth
	20_72	9.12	4.89	0.025	*PDCD6/SLC9A3*	[136, 137]; human and mice, goat; cell membrane repair and apoptosis, diet induced changes in the rumen
	21_13	8.56	5.11	0.024	*MCTP2*	[138]; human; adiposity and obesity
	18_18	7.90	6.54	0.022	*CBLN1/C18H16orf78/ZNF423*	[139, 140]; rat, cattle; appetite regulation, feed efficiency and growth
	1_108	7.64	5.29	0.021	*PPM1L*	[132]; mouse; obesity, metabolic syndrome, and growth
	1_133	7.33	4.79	0.020	*NCK1*	[133]; mouse; glucose tolerance and insulin resistance
Hereford	X_113	9.50	19.66	0.046	*MAGEB16/LOC786694*	[141]; rat; diet induced differential expression and potential risk for carcinogenesis
	X_105	6.76	13.00	0.032	*MAOA/MAOB*	[142]; human; obesity
	20_5	5.96	−3.76	0.028	*STC2/LOC101903982/ERGIC1/RPL26L1/ATP6V0E1/CREBRF/BNIP1/NKX2-5*	[143, 144]; human, mouse; adiposity and obesity, skeletal muscle development
	14_6	5.44	9.24	0.025	*LOC104973978/FAM135B/LRP12/DPYS*	[11, 145, 146]; cattle, chicken, human; feed efficiency and growth, obesity, growth retardation
	19_56	5.26	6.61	0.024	*MGAT5B/MFSD11/SRSF2/METTL23/JMJD6*	[68, 147–149]; mice, human; glycosylation, interaction with genes differentially expressed in brown adipose, promoting adipogenic differentiation.
	22_11	4.98	3.98	0.023	*ITGA9*	[54]; cattle; feed efficiency
	8_2	4.63	−3.87	0.021	*LOC101904351/LOC520638/AADAT*	[81, 111]; chicken, seabass; feed efficiency and growth
	26_19	4.55	6.99	0.021	*GOLGA7B/CRTAC1*	[150, 151]; buffalo, mice; dairy traits, lateral olfactory tract formation
	X_145	4.40	−3.90	0.020	*ANOS1*	[152, 153]; human; puberty timing, serum and free testosterone
SimAngus	14_25	6.89	−2.73	0.023	*LYN/RPS20/MOS/PLAG1/CHCHD7/SDR16C5/SDR16C6/PENK*	[154–156]; bovine, human; birthweight and stature, height
	14_24	6.61	2.61	0.022	*LOC104974018/XKR4/TGS1/LYN/RPS20/MOS/PLAG1/CHCHD7/SDR16C5/SDR16C6/PENK*	[154–156]; bovine, human; birthweight and stature, height
	17_18	5.15	−1.91	0.017	*MAML3*	[157]; pig; downregulated in obese pigs
	20_5	5.13	2.39	0.016	*STC2/LOC101903982/ERGIC1/RPL26L1/ATP6V0E1/CREBRF/BNIP1/NKX2-5*	[143, 144]; human, mouse; adiposity and obesity, skeletal muscle development
	X_148	5.10	−2.33	0.016	*NLGN4X*	[158, 159]; human; sex-biased exon usage, muscle and development pathways
	4_10	4.94	5.70	0.016	*HEPACAM2*	[160]; human; mitosis
	28_1	4.64	−1.74	0.015	*TAF5L/URB2*	[161, 162]; human; diabetes, fasting insulin levels
	6_39	4.63	1.88	0.015	*LOC101904320/LCORL/NCAPG*	[163, 164]; cattle; feed efficiency and growth traits, body frame size
	14_26	4.51	2.08	0.014	*FAM110B/UBXN2B/NSMAF/TOX*	[165]; cattle; Brahman puberty traits
	13_50	4.51	1.84	0.014	*BMP2*/*LOC104973806/LOC104973807/LOC101904237/LOC104973808/LOC104973809*	[166]; pig; loin muscle area, body size, and structure traits
	13_16	4.32	−2.11	0.014	*ITIH5/LOC104973716/LOC101903501/SFMBT2*	[167, 168]; human; fasting insulin and BMI
	27_22	4.32	−3.45	0.014	*SGCZ*	[41]; human; obesity-related traits

Investigation of MMWT in Angus using the 778K genotype set revealed evidence for eight large-effect QTL (PVE ≥ 2.0%) distributed across five autosomes (Table 6). The lead SNP defining the largest-effect QTL on BTA7 (7_24 Mb) explained 4.6% of the variance in MMWT, with the positional candidate gene *ACSL6* directly underlying the QTL signal. This result is concordant with the previous Bayesian study employing 1 Mb windows, in which *ACSL6* was implicated for QTL underlying both DMI and MMWT [13]. In contrast, we place the Angus DMI QTL signal approximately 3 Mb away from *ACSL6*, and approximately 2 Mb from an ADG QTL (Tables 4, 5 and 6). It should also be noted that the largest-effect QTL detected for MMWT in Angus was also proximal to a SimAngus ADG QTL (7_26 Mb; Table 5). While *ACSL6* has been identified as a strong positional candidate gene for feed efficiency and growth in Angus [13], it has also been implicated in diabetic myocardial metabolism in rodents, with evidence for *ACSL6* being an insulin-regulated gene [129]. Among the eight large-effect MMWT QTL detected in Angus, three were located on BTA1 (1_98 Mb; 1_108 Mb; 1_133 Mb). Positional candidate genes (*MYNN*, *ACTRT3*; Table 6) which were proximal to the lead SNP at 98.5 Mb on BTA1 have been implicated in obesity and diabetes traits in humans and mice, respectively [130, 131]. Moreover, two proximally relevant positional candidate genes were also suggested by the locations of the lead SNPs for Angus MMWT QTL detected near 108 Mb (*PPM1L*) and 133 Mb (*NCK1*) on BTA1, with *PPM1L* previously being associated with mouse obesity, metabolic syndrome, and growth [132], and *NCK1* associated with glucose tolerance and insulin resistance in mice [133]. Other large-effect MMWT QTL were detected on BTA7 (7_0 Mb), BTA20 (20_72 Mb), BTA21 (21_13 Mb), and BTA18 (18_18 Mb; Table 6). Several positional candidate genes were proximal to the lead SNP defining a second BTA7 QTL (7_0 Mb; Table 6) for MMWT including *CNOT6*, which together with a related paralog, *CNOT6L*, has been associated with the regulation of human cell growth and survival [134, 135]. Additionally, at least four unannotated genes are also in close physical proximity to the lead SNP on BTA7, including an olfactory receptor like gene, two loci with similarity to *CLEC7A* (a C-type lectin domain superfamily member), one uncharacterized locus, and *FLT4* which encodes a tyrosine kinase receptor for vascular endothelial growth factors C and D (Table 6).

Three positional candidate genes were identified for the Angus MMWT QTL detected on BTA20 (20_72 Mb) and BTA21 (21_13 Mb) (Table 6). On BTA20, the lead SNP (71.9 Mb) was proximal to *PDCD6* and *SLC9A3*, which have been associated with muscle cell membrane repair in mice, and diet induced changes of the caprine rumen, respectively [136, 137]. The lead SNP for the Angus MMWT QTL on BTA21 (21_13 Mb; Table 6) was adjacent to *MCTP2* (13.24 Mb), which is known to be associated with human adiposity and obesity traits [138]. Additionally, two positional candidate genes for the MMWT QTL on BTA18 (18_18 Mb) have been associated with appetite regulation in the rat (*CBLN1*), and feed efficiency and growth in cattle (*ZNF423*) [139, 140]. A final investigation of all Illumina 778K markers that met or exceeded the Wellcome Trust significance threshold [27] revealed additional marker-based support for seven of the eight large-effect, and at least eight moderate-effect (1.0% ≤ PVE ≤ 2.0%) MMWT QTL (i.e., 7_68 Mb; 8_80 Mb; 9_91 Mb; 10_44 Mb; 14_68 Mb; 17_35 Mb; 17_41 Mb; 19_42 Mb; Additional file 1). The directions of the marker effects were also consistently positive for all eight large-effect Angus MMWT QTL (Table 6), which was a unique observation among the three populations and traits analyzed in this study. The MAFs for all lead SNPs defining the eight large-effect MMWT QTL in Angus ranged from 0.06 (BTA18_18 Mb) to 0.20 (BTA7_24 Mb), with six of the eight QTL having lead SNPs possessing MAFs ≥ 0.11. Reduction of the marker set from 778K to 50K revealed four markers that met or exceeded the Wellcome Trust significance threshold [27], but none of the markers identified the genomic regions harboring the eight largest-effect QTL signals detected in the 778K analysis (Table 6). Among the top 100 ranked 50K markers, there was no evidence to support the eight largest-effect Angus MMWT QTL.

Analysis of MMWT in Hereford using the 778K genotypes revealed evidence for nine large-effect QTL (PVE ≥ 2.0%) distributed across six autosomes and BTAX (Table 6). The two largest-effect QTL were detected on BTAX (X_113 Mb; X_105 Mb), with lead SNPs (112.90 Mb; 105.46 Mb) that were estimated to explain approximately 4.6% and 3.2% of the variance in MMWT. The location of the lead SNP near 113 Mb was upstream of the transcriptional start site of *MAGEB16*, which is differentially expressed under high and low protein diets, and is associated with pathways related to cancer [141]. The primary positional candidate genes underlying the lead SNP on BTAX near 105 Mb were *MAOA* and *MAOB*, which are associated with human obesity [142]. Moreover, the proposed mechanism of action by which *MAOA* and *MAOB* genotypes influence obesity relates to dopamine bioavailability, which is implicated in appetite regulation [142]. We also detected a MMWT QTL on BTA20 (20_5 Mb; lead SNP 4.90 Mb), which ranked third in our genome-wide analysis (PVE = 2.8%), and for which we identified several biologically important positional candidate genes including *STC2* and *LOC101903982* (*SYNPO2* like; Table 6). Notably, *STC2* is an endoplasmic reticulum stress response gene that is associated with adiposity and obesity in nondiabetic humans [143], while *SYNPO2* plays a role in early skeletal muscle development (myofibrillogenesis), and because of its association with myopathy-related proteins, *SYNPO2* is considered a candidate gene for muscle disease [144]. Beyond *STC2*, several additional genes encoding endoplasmic reticulum-related proteins (http://www.genecards.org) were also noted proximal to the MMWT QTL on BTA20 (20_5 Mb) including *ERGIC1*, *CREBRF*, and *BNIP1* (Table 6).

Interestingly, most of the primary positional candidate genes for a large-effect QTL on BTA14 (14_6 Mb; lead SNP 6.02 Mb; PVE = 2.5%) have been associated with aspects of feed efficiency and growth or obesity (Table 6) across three vertebrate species [11, 145, 146]. Specifically, *FAM135B* has been associated with residual average daily gain (RADG) in SimAngus [11], and *LRP12* is differentially expressed in the adipose tissue of divergently selected (lean versus fat) broiler lines [145] (Table 6). The biological significance of the positional candidate gene *DPYS* for the BTA14 (14_6 Mb) QTL stems from the fact that a deficiency in the enzyme encoded by this gene (dihydropyrimidinase) has been proposed to modulate growth retardation, failure to thrive, and other disadvantageous phenotypes in humans [146]. An evaluation of positional candidate genes colocalizing to the Hereford MMWT QTL on BTA19 (PVE = 2.4%; lead SNP 55.67 Mb) suggested that pleiotropic QTL influence both DMI and MMWT in Hereford (Tables 4 and 6), or that independent but proximal causal mutations influence the traits. While the lead SNPs identifying these QTL were not identical, they were proximally located (≈ 141 kb), implicating *MGAT5B* as a positional candidate gene for both DMI and MMWT (Tables 4 and 6). While *MGAT5B* is involved in the biosynthesis of N-linked and *O*-mannosyl-linked glycosylation [62, 68], at least one prior investigation of this locus was catalyzed by the supposition that inactivation of the glycosyltransferases comprising the *O*-mannosyl processing pathway leads to human congenital disorders characterized by muscular atrophy with neuronal defects [68]. Additional positional candidate genes flanking the MMWT QTL on BTA19 include *METTL23* and *JMJD6* (Table 6). The protein encoded by *METTL23* has methyltransferase activity and regulates a pathway underlying human cognition as well as *GABPA* function [147]. *GABPA*, which encodes a mitochondrial biogenesis and maintenance protein, was recently shown to be differentially expressed in the brown adipose tissue of mice subject to exercise, dietary restriction, and ephedrine treatment; with all such treatments resulting in weight loss as compared to controls [148]. Moreover, *JMJD6*, which encodes a nuclear protein involved in histone modification, transcription, RNA processing, and tissue development, was recently shown to have multiple roles in promoting adipogenic differentiation in mouse cells [149]. A large-effect MMWT QTL supported by 17 SNPs also was detected on BTA22 (lead SNP 11.03 Mb; PVE = 2.3%), with *ITGA9* implicated as a primary positional candidate gene. Notably, *ITGA9* has recently been associated with RFI in dairy cattle [54].

For U.S. Hereford cattle, our analysis of MMWT again revealed the potential for proximal but independent causal mutations, or a pleiotropic QTL on BTA8 (8_2Mb) that influences ADG and MMWT (Table 5, Table 6), but that may also influence DMI (Additional file 1). While the lead SNP defining the Hereford MMWT QTL differed among the three traits, all were located upstream of the *AADAT* transcriptional start site, with the lead SNP for the MMWT QTL being most proximal to this positional candidate gene (BTA8 at 1.90 Mb). While we did not detect large-effect MMWT QTL for Hereford on BTA7 (7_93 Mb) or BTA18 (18_63 Mb) as previously described [13], we did replicate the QTL on BTA20 [13]; the only difference being that we defined QTL position (20_5 Mb) by rounding the position of the lead SNP (4.90 Mb) to the nearest Mb. One plausible explanation for the differences noted between the present study and a Bayesian 1 Mb window analysis [13], was our inclusion of additional fixed effects within the mixed model (See Methods). Moreover, we also detected two large-effect MMWT QTL (26_19 Mb; lead SNP 19.13 Mb; PVE = 2.1%; X_145 Mb; lead SNP 145.33 Mb; PVE = 2.0%) which were not previously detected [13] (Table 6). The two primary positional candidate genes *GOLGA7B* and *CRTAC1* implicated by the lead and supporting SNPs on BTA26 (26_19 Mb) have been associated with dairy production traits in buffaloes, and lateral olfactory tract formation in mice [150, 151]. Investigation of the lead and supporting SNPs (Additional file 1) defining a third large-effect QTL detected on BTAX near 145 Mb identified *ANOS1* (i.e., previously *KAL1*) as the primary positional candidate gene (Table 6). *ANOS1* has been associated with puberty timing in humans and serum testosterone levels in men; with this gene encoding an extracellular matrix protein implicated in the embryonic migration of gonadotrophin releasing hormone and olfactory-related neurons [152, 153]. The MAFs for all lead SNPs defining the nine large-effect MMWT QTL in Hereford ranged from 0.01 (BTAX_113 Mb) to 0.39 (BTA20_5 Mb), with seven of the nine QTL having lead SNPs possessing MAFs > 0.05 (Additional file 1). Reduction of the marker content from 778K to 50K revealed 11 markers that met or exceeded the Wellcome Trust significance threshold [27], and included four of the nine largest-effect QTL that were detected in the 778K analysis (≤ 1 Mb from X_113 Mb; X_105 Mb; 20_5 Mb; 22_11 Mb; Table 6). Further investigation of the 100 top-ranked 50K markers revealed evidence supporting one additional large-effect QTL detected in the 778K analysis (≤ 1 Mb from 19_56 Mb; Table 6).

Analysis of the 778K genotypes for U.S. SimAngus revealed evidence for two large-effect (PVE ≥ 2.0%) and 10 moderate-effect (1.0% > PVE < 2.0%) MMWT QTL (Table 6). The lead SNPs identifying the two large-effect QTL on BTA14 (14_25 Mb, PVE = 2.3%; 14_24 Mb, PVE = 2.2%) were ≤ 590 kb apart (at 24.9 Mb and 24.3 Mb). However, both QTL were supported by additional SNPs, including some that were located ≥ 1 Mb apart (Additional file 1), suggesting that independent causal mutations influence MMWT in this region of BTA14 in SimAngus. At least eight biologically relevant positional candidate genes were identified within the QTL intervals spanning 14_25 Mb and 14_24 Mb (Table 6), including *LYN*, *RPS20*, *MOS*, *PLAG1*, *CHCHD7*, *SDR16C5*, *SDR16C6* and *PENK*. The genomic region harboring these genes is of particular interest considering previous associations with birth weight in Nellore cattle (*LYN*, *RPS20*, *MOS*, *PLAG1*, *SDR16C5*, *SDR16C6*, *PENK*), variation in adult human height (*TGS1*, *LYN*, *RPS20*, *MOS*, *PLAG1*, *CHCHD7*, *SDR16C5*, *SDR16C6*, *PENK*), and variation in body stature (hip-height) among beef and dairy cattle (*PLAG1*, *CHCHD7*, *SDR16C5*, *SDR16C6*) [154–156]. A moderate-effect MMWT QTL detected on BTA17 (17_18 Mb; lead SNP 18.04 Mb; PVE = 1.7%) suggests a positional candidate gene (*MAML3*) that was associated with the degree of obesity (obesity index) in pigs during an expression QTL analysis [157]. As in Hereford, we also detected a MMWT QTL in SimAngus on BTA20 (20_5 Mb; lead SNP 4.91 Mb; PVE = 1.6%; Table 6). The genomic location of this QTL physically overlaps between the two populations in this study, including some concordant supporting SNPs between the SimAngus MMWT QTL and the Hereford MMWT QTL (Additional file 1) whereby *STC2* was implicated as the primary positional candidate gene (Table 6). This result is only partially concordant with the results of the Bayesian study employing non-overlapping 1 Mb windows, in which *STC2* was found to underlie a large-effect QTL for MMWT and RFI in Hereford, but was not implicated in MMWT in SimAngus [13]. While the previous study reported a SimAngus MMWT QTL on BTA20 (20_6 Mb), the identified 1 Mb window was not concordant with the genomic positions of the lead and supporting SNPs from our analysis (lead SNP position = 4.91 Mb; range of supporting SNP positions = 4.87 Mb to 4.91 Mb, *n* = 7 supporting SNPs).

One moderate-effect MMWT QTL was detected on BTAX (X_148 Mb; lead SNP 147.54; PVE 1.6%) in SimAngus, with *NLGN4X* directly underlying both the lead and the supporting SNPs (Table 6). *NLGN4X* encodes a neuronal cell surface protein that is a member of the type-B carboxylesterase/lipase protein family, and has been observed to exhibit sex-biased exon usage across multiple developmental time points in humans, but has no known association with feed efficiency or growth [158]. However, *NLGN4X* knockdown in human neural stem cells significantly altered the expression of genes comprising several biologically relevant ontology groups including organ development (GO:0048513), muscle organ development (GO:0007517), muscle contraction (GO:0006936), tissue development (GO:0009888), regulation of cell morphogenesis (GO:0022604), epithelial cell differentiation (GO:0030855), and epithelium development (GO:0060429) [159]. The positional candidate gene *HEPACAM2*, which functions as a mitotic kinetics regulator, and likely also as a tumor suppressor gene, was found to directly underlie a moderate-effect SimAngus MMWT QTL on BTA4 (4_10 Mb; lead SNP 10.41 Mb; PVE 1.6%; Table 6), with knockdown of *HEPACAM2* expression in human cell lines resulting in mitotic arrest, disorganized spindles, and scattered chromosomes [160]. Relatively few positional candidate genes were identified for five additional moderate-effect MMWT QTL that were detected in SimAngus on BTA28 (28_1 Mb; lead SNP 0.68 Mb; PVE = 1.5%), BTA6 (6_39 Mb; lead SNP 39.25 Mb; PVE = 1.5%), BTA14 (14_26 Mb; lead SNP 26.28 Mb; PVE = 1.4%), and BTA13 (13_50 Mb and 13_16 Mb; lead SNPs 49.96 Mb and 16.36 Mb; PVE = 1.4%; See Table 6). On BTA28, the protein coding genes *TAF5L* and *URB2* were most proximal to both the lead and the supporting SNPs defining this QTL. Human genetic variation in *TAF5L*, which is involved in myogenic transcription and differentiation, has been associated with risk for type-1 diabetes, while rare coding variants in *URB2*, a ribosome biogenesis gene, have been associated with fasting insulin levels in humans [161, 162]. Relevant to feed efficiency and growth QTL recently detected and described [13], *LOC101904320*, *LCORL*, and *NCAPG* were proximal to the MMWT QTL on BTA6 (6_39 Mb; Table 6). Both the lead and all supporting SNPs were located in a noncoding intergenic region flanking all three positional candidate genes. This result is interesting because *LCORL* and *NCAPG* have recently been reported to underlie a MMWT QTL in Angus that spanned two contiguous 1 Mb windows identified by a Bayesian analysis (6_38 Mb; 6_39 Mb), and this genomic region is also known to harbor feed efficiency, growth, and carcass QTL (http://www.animalgenome.org/cgi-bin/QTLdb/BT/index) [13, 163, 164]. Four relevant positional candidate genes (*FAM110B*, *UBXN2B*, *NSMAF*, *TOX*) were identified for the MMWT QTL on BTA14 (14_26 Mb), with the lead SNP located in an intron of *UBXN2B*, a protein coding gene involved in endoplasmic reticulum biogenesis (Table 6). However, it should also be noted that *FAM110B*, *NSMAF*, and *TOX* have all previously been associated with puberty related traits in Brahman cattle, including age at formation of the first *corpus luteum*, and age at which scrotal circumference was ≥ 26 cm [165].

For the SimAngus MMWT QTL on BTA13 near 50 Mb, the most proximal protein coding gene with reference annotation was *BMP2*, while *ITIH5* was determined to directly underlie the QTL near 16 Mb. We also noted that the transcriptional start site of *SFMBT2* was proximal to both the lead and supporting SNPs that defined this QTL (13_16 Mb). Relevant to feed efficiency and growth, *BMP2* has previously been associated with loin muscle area, body size, and structural traits in pigs, while *ITIH5* and *SFMBT2* have been associated with fasting insulin levels and BMI in humans, respectively [166–168]. Finally, the lead and supporting SNPs underlying the QTL on BTA27 (27_22 Mb) were found within an intron of *SGCZ* (lead SNP 21.70 Mb), which encodes a protein that bridges the inner cytoskeleton and the extra-cellular matrix, and has been associated with obesity-related traits in humans [41]. The MAFs for all lead SNPs defining the MMWT QTL detected in SimAngus ranged from 0.04 (4_10 Mb) to nearly 0.50 (13_50 Mb, MAF = 0.497), with 10 of the 12 QTL having lead SNPs possessing MAFs ≥ 0.15 (Additional file 1). Following a reduction in marker content from 778K to 50K, four markers met the Wellcome Trust significance threshold [27] for MMWT in SimAngus and supported the two largest-effect MMWT QTL detected in the 778K analysis (≤ 1 Mb from 14_25 Mb and 14_24 Mb), and three moderate-effect QTL (≤ 1 Mb from 6_39 Mb, 13_50 Mb, and 13_16 Mb). Evaluation of the 100 top-ranked 50K markers provided support for all of the other moderate-effect SimAngus MMWT QTL, with the exception of BTA4 (4_10 Mb).

## Conclusions

We present evidence for both large (PVE ≥ 2.0%) and moderate-effect QTL (1.0% ≤ PVE ≤ 2.0%) that influence RFI, DMI, ADG, and MMWT in U.S. Angus, Hereford, and SimAngus beef cattle. Collectively, the positional candidate genes implicated by the QTL analyses for these populations suggest that feed efficiency and growth-associated loci are likely to be conserved across vertebrate species. Moreover, among the detected feed efficiency and growth QTL, we frequently observed positional candidate genes that had previously been associated with obesity-related traits or metabolic syndrome, biological aspects of adiposity, diabetes-related traits, and feed efficiency and growth traits across a variety of vertebrate species (humans, mice, rats, pigs, chickens, fish), which suggests both a relationship among phenotypes (i.e., feed efficiency, metabolic syndrome and adiposity) and a conserved biological system underlying feed intake and efficiency. Moreover, we detected 14 QTL regions within and between populations that ranged from being physically proximal (≤ 3 Mb) to fully overlapping for RFI, DMI, ADG, and MMWT, suggesting the existence of pleiotropy, proximal but independent causal mutations influencing one or more of these traits, and some multi-breed QTL (Additional file 1). For example, one such 3 Mb QTL interval (19_54 Mb to 19_57 Mb) was implicated in every analyzed trait (i.e., Hereford: RFI, DMI, MMWT; SimAngus: ADG). Finally, our comparison of the detection resolution limits for the 50K versus the 778K assays is important, particularly since many previous analyses have been performed using 50K genotypes, with the results used to catalyze genetic progress in U.S. beef and dairy cattle. Herein, we demonstrate that while the 50K and the 778K produce very similar heritability estimates for RFI, DMI, ADG, and MMWT in our populations, some large and moderate-effect QTL go undetected in the 50K analysis, potentially reducing the opportunities for causal variant discovery. Therefore, additional QTL of moderate to large-effect will undoubtedly be discovered in historic data sets by imputing the data from 50K to 778K or beyond, and repeating previously performed analyses, which are also likely to produce further evidence for pleiotropy or multi-population segregation.

## Methods

All feed efficiency and growth data were either collected by commercial producers or under the approval from the University of Missouri (ACUC Protocol 7505) or the University of Illinois at Champaign-Urbana (IACUC Protocols 06091 and 09078) Animal Care and Use Committees.

### Cattle populations, phenotypes, and genotypes

All study animals, genotyping, and methods related to the ascertainment of phenotypes of interest such as ADG, daily average DMI, and MMWT, measured in Angus, Hereford, and SimAngus cohorts, were recently described [13]. Likewise, residual feed intake (RFI) was estimated by including partial linear regressions on ADG and MMWT in the model used to analyze DMI [13]. Using the available BovineHD genotypes previously described for the Angus, Hereford, and SimAngus cohorts, we conducted sample filtering by call rate (< 0.90), SNP filtering by call rate (< 0.85), and MAF filtering (< 0.001) as described by Saatchi and colleagues [13]. Details regarding the cattle included in the present study were as follows: (A) 1572 U.S. Angus steers were available for the analysis of ADG and MMWT, whereas 706 purebred Angus steers were used to analyze DMI and RFI (See Statistics for further details); (B) 850 U.S. Hereford cattle (826 steers, 24 heifers) were available for the analysis of ADG, DMI, MMWT, and RFI; (C) 1465 U.S. SimAngus steers were available for the analysis of ADG, DMI, MMWT, and RFI. For the 778K analyses, there were 722,716 SNPs for Angus, 659,688 SNPs for Hereford and 653,132 SNPs for SimAngus. All 50K analyses were performed following a population-specific reduction in marker content from the filtered 778K to 50K density resulting in 47,582, 48,728, and 45,926 available markers for Angus, Hereford, and SimAngus respectively.

### Statistics

Genome-wide association analyses were performed using a mixed linear model, implemented in EMMAX [24, 169–171], and were executed in Python as well as the SVS environment (Golden Helix, Version 7.7.6), as previously described [169, 170]. The mixed model used in this study can be generally specified as: *y* = *Xβ* + *Zu* + *ϵ*, where *y* is a *n* × 1 vector of the observed phenotypes, *X* is a *n* × *q* matrix of fixed effects, *β* is a *q* × 1 vector representing the coefficients of fixed effects, and *Z* is a *n* × *t* matrix relating instances of the random effect to the specified phenotypes [169, 170] (http://doc.goldenhelix.com/SVS/8.2.1/mixed_models.html). Moreover, we assume that *Var*(*u*) = *σ*_*g*_^2^ *K* and *Var*(*ϵ*) = *σ*_*e*_^2^ *I*, such that *Var*(*y*) = *σ*_*g*_^2^ *ZKZ* ^'^ + *σ*_*e*_^2^ *I*, but in the present study, *Z* is simply the identity matrix *I*, and *K* is a kinship matrix among all genotyped samples. To solve the mixed model equations using a generalized least squares approach, the variance components (*σ*_*g*_^2^ and *σ*_*e*_^2^) must be estimated as previously described [24, 169, 171] (http://doc.goldenhelix.com/SVS/8.2.1/mixed_models.html). We used the REML-based EMMA approach to estimate variance components [24, 171], with stratification accounted for and controlled using the genomic kinship matrix [23] computed from either the filtered Illumina 778K or 50K genotypes. Models were evaluated using phenotypes and available data previously described [13], with final models parameterized to include the following: (A) Angus RFI included DMI as the dependent variable, with date of birth (DOB), contemporary group (CG), days on feed (DOF), ADG, and MMWT as covariates (*n* = 706 observations); Angus DMI included DOB, CG, and DOF as covariates (*n* = 706 observations); Angus ADG included DOB (mean for missing values), percent Angus (i.e., not all were purebred), CG, and DOF as covariates (*n* = 1572 observations); Angus MMWT included DOB (mean for missing values), percent Angus (i.e., not all purebred), CG, and DOF as covariates (*n* = 1572 observations); Briefly, 706 purebred Angus were used for final RFI and DMI analyses because they possessed complete information regarding DOB, and were all fed a uniform diet; (B) Hereford RFI included DMI as the dependent variable, with DOB, breed composition (i.e., not all were purebred), CG, sex, ADG, and MMWT as covariates (*n* = 846 observations); Hereford DMI included DOB, breed composition, CG, and sex as covariates (*n* = 846 observations). Hereford ADG included DOB, breed composition, CG, and sex as covariates (*n* = 849 observations); Hereford MMWT included DOB, breed composition, CG, and sex as covariates (*n* = 849 observations); (C) SimAngus RFI included DMI as the dependent variable, with DOB, breed composition, ranch, pen, experimental year, nutritional treatments (diet), DOF, slaughter group (SG), ADG, and MMWT as covariates (*n* = 1217 observations); SimAngus DMI included DOB, breed composition, ranch, pen, experimental year, nutritional treatments (diet), DOF, and SG as covariates (*n* = 1218 observations); SimAngus ADG included DOB, breed composition, ranch, pen, experimental year, nutritional treatments (diet), DOF, and SG as covariates (*n* = 1237 observations); SimAngus MMWT included DOB, breed composition, ranch, pen, experimental year, nutritional treatments (diet), DOF, and SG as covariates (*n* = 1238 observations). All covariates were specified and treated as previously described [24]. Prior to analysis, all array genotypes were recoded as 0, 1, or 2, based on the count of the minor allele for that animal at each SNP marker. For 778K and 50K analyses, markers were ranked by *P*-value and PVE. Bovine 778K QTL were defined by ≥ 2 markers with MAF ≥ 0.01 (i.e., a lead SNP plus at least one supporting SNP within 1 Mb) which also met a nominal significance (*P* ≤ 5e-05) [27] and PVE [24, 25, 169] threshold (PVE ≥ 1.0%), and those QTL were evaluated, reported and discussed. However, a few putative QTL signals were presented for which only 1 lead SNP met all reporting criteria, and with one or more supporting SNPs falling below the nominal significance threshold (i.e., 28_45 Mb SimAngus RFI − *TMEM72*, *P* < 1e-04). For the 50K analyses, the following criteria were used to evaluate whether a 778K QTL was replicated: Among the top 100 ranked 50K SNPs, we required ≥ 1 SNP to be within 1 Mb of the 778K lead SNP.

## Additional file

Additional file 1: Summary data for all lead and supporting SNPs from the 778K analyses for RFI, DMI, ADG, and MMWT, including dbSNP identifiers and QTL coordinates. (XLSX 710 kb)
